# Functional analysis of bovine TLR5 and association with IgA responses of cattle following systemic immunisation with H7 flagella

**DOI:** 10.1186/s13567-014-0135-2

**Published:** 2015-02-19

**Authors:** Amin Tahoun, Kirsty Jensen, Yolanda Corripio-Miyar, Sean P McAteer, Alexander Corbishley, Arvind Mahajan, Helen Brown, David Frew, Aude Aumeunier, David GE Smith, Tom N McNeilly, Elizabeth J Glass, David L Gally

**Affiliations:** Division of Immunity and Infection, The Roslin Institute and R(D)SVS, The University of Edinburgh, Easter Bush, Midlothian, EH25 9RG UK; Faculty of Veterinary Medicine, Kafrelsheikh University, 33516 Kafr el-Sheikh, Egypt; Moredun Research Institute, Pentlands Science Park, Bush Loan, Penicuik Edinburgh, EH26 OPZ UK; Institute of Infection, Immunity & Inflammation, Glasgow Biomedical Research Centre, University of Glasgow, Glasgow, G12 8TA UK

## Abstract

Flagellin subunits are important inducers of host immune responses through activation of TLR5 when extracellular and the inflammasome if cytosolic. Our previous work demonstrated that systemic immunization of cattle with flagella generates systemic and mucosal IgA responses. The IgA response in mice is TLR5-dependent and TLR5 can impact on the general magnitude of the adaptive response. However, due to sequence differences between bovine and human/murine TLR5 sequences, it is not clear whether bovine TLR5 (bTLR5) is able to stimulate an inflammatory response following interaction with flagellin. To address this we have examined the innate responses of both human and bovine cells containing bTLR5 to H7 flagellin from *E. coli* O157:H7. Both HEK293 (human origin) and embryonic bovine lung (EBL) cells transfected with bTLR5 responded to addition of H7 flagellin compared to non-transfected controls. Responses were significantly reduced when mutations were introduced into the TLR5-binding regions of H7 flagellin, including an R90T substitution. In bovine primary macrophages, flagellin-stimulated CXCL8 mRNA and secreted protein levels were significantly reduced when TLR5 transcript levels were suppressed by specific siRNAs and stimulation was reduced with the R90T-H7 variant. While these results indicate that the bTLR5 sequence produces a functional flagellin-recognition receptor, cattle immunized with R90T-H7 flagella also demonstrated systemic IgA responses to the flagellin in comparison to adjuvant only controls. This presumably either reflects our findings that R90T-H7 still activates bTLR5, albeit with reduced efficiency compared to WT H7 flagellin, or that other flagellin recognition pathways may play a role in this mucosal response.

## Introduction

Flagella have been shown to play a significant role in bacterial pathogenesis, primarily through their function as motility organelles, but also as adhesins and as pro-inflammatory agonists. As a consequence, flagella have been trialled as vaccine antigens in a number of species [1-5] and it is evident that flagellins promote specific immune responses and may increase the magnitude of the response, functioning as an adjuvant for the presentation of heterologous antigens [6,7]. It has been demonstrated in cattle that systemic vaccination with H7 flagella leads to the production of IgA and IgG1 against FliC_H7_ with both IgA and IgG1 detected at the mucosal surface [3,8,9]. Toll like receptors (TLRs) are crucial components that allow recognition of microbial associated molecular patterns (MAMPs), including Lipid A of LPS, lipoteichoic acid, peptidoglycan, certain nucleic acids and flagellin [10,11]. TLRs are a family of transmembrane proteins, each consisting of a Leucine-rich extracellular domain (ectodomain) that recognizes distinct MAMPs and hence is variable between different TLRs. Most TLRs form dimers following MAMP binding and some TLRs can function as heterodimers, for example TLR2 makes a heterodimer with TLR6 to sense lipoteichoic acid and a heterodimer with TLR1 to sense lipid-protein combination [12].

TLR5 recognises the flagellin monomer [13,14] and they are considered to form a TLR5:flagellin complex with a 2:2 stoichiometry [15]. TLRs have an intracellular domain (endodomain) that is relatively conserved between the different TLRs including the presence of a toll/interleukin-1 (TIR) region that contains specific amino acids that are phosphorylated upon MAMP binding and can then interact with different adaptor proteins leading to signalling cascades resulting in pro-inflammatory cytokine release [16,11]. In terms of flagellin, it is evident that specific residues within the more conserved D1 domains are required for binding to the TLR5 ectodomain, with the more variable D2 and D3 regions responsible for the antigenic variability of flagellins [17]. While the D0 and D1 domains of flagellin are relatively conserved, variation in these regions has been shown to limit innate responses to flagellin expressed by α and ε Proteobacteria, including *Helicobacter pylori* [18,19].

Recent research in mice has indicated that induction of IgA following systemic immunization with flagellin from *Salmonella enterica serovar* Typhimurium is considered to be dependent on the capacity of monomeric flagellin to stimulate toll-like receptor 5 (TLR5) signalling in specific intestinal dendritic cells [20]. Another study in mice has also shown that the magnitude of the response to flagellin as an antigen is also TLR5-dependent [21]. There is 79% amino acid homology between the bovine (NP_001035591.1) and human (NP_003259.2) TLR5 sequences. In cattle (*Bos taurus*), there are specific changes in both the extracellular domain and the TIR domain in TLR5 in comparison to other mammalian sequences [22]. For example, codon S268 has been positively selected in the artiodactyl clade [22] and is within the LRR9 loop region identified by Yoon et al. [17] as being particularly important in the TLR5 interaction with the N- and C-terminal helices of the flagellin D1 domain. Variation in the human and murine amino acids at this site (TLR5 268) has also been shown to at least partially account for differences between these species in their interactions with flagellins [23]. With the addition of further artiodactyla TLR5 sequences we have identified a further potentially functional change in bovine TLR5 at position 798 in the TIR region, which in most species including humans and mice is a tyrosine, but in cattle and other ruminants is a Phenylalanine. It has been suggested that Tyr798 plays an important role in TLR5 signaling as mutation of murine TLR5 Tyr798 to Leu798 resulted in negligible capacity to signal in response to flagellin [23]. Very recent work demonstrated that bovine TLR5 transfected into HEK293T cells did not appear to signal. However there was some indication of P38 phosphorylation in bovine macrophages following addition of flagella although there was no indication as to whether this was TLR5-dependent [24]. Taken together, it is unclear whether TLR5 is functionally important in the recognition of flagellin in cattle.

Nonetheless, systemic immunization in cattle with H7 flagellin induces IgA responses [3,8] in line with similar murine responses to flagellin that were dependent on TLR5 [20,21,23]. This might indicate that these bovine IgA responses are also TLR5-dependent and that bovine TLR5 is functional despite variation in the ectodomain and the TIR domain. To test this we have transfected bovine TLR5 (bTLR5) clones into both human HEK293 and bovine EBL cells and analysed responses following stimulation with wild type H7 and with flagellins engineered to contain site-specific mutations in the TLR5-binding domains. We also investigated gene expression and CXCL8 secretion from bovine peripheral blood monocyte-derived macrophages following addition of H7 flagellin, with and without siRNA knock-down of bovine TLR5 expression. Finally, immunization studies were carried out in cattle comparing stimulation of mucosal humoral responses following systemic delivery of H7 flagella and a variant with reduced capacity to stimulate TLR5.

## Materials and methods

### Flagella expression and purification

H7 flagella or engineered variants were expressed from *E. coli* O157 TUV93-0 Δ*fliC* transformed with pEW7 (wild type *fliC*_*H7*_) or plasmid-encoded site-directed mutants (Table 1). Flagella were purified by shearing for all the in vitro experiments according to published procedures and by acid de- and re-naturation for the calf immunization trials [3,8,25]. In brief, one colony was grown overnight on motility agar at 30 °C. 10 μL agar plugs from the edge of the motility circle were then inoculated overnight in LB broth with ampicillin (100 μg/mL). The next day this was diluted 1:100 into LB with ampicillin and 1 mM IPTG added when the culture reached OD_600_ = 0.5, and the cultures left to grow at 30 °C for 4-5 h (OD_600_ > 2). The bacteria were harvested at 4100 × *g* at 4 °C for 30 min. The supernatants were discarded and the pellets suspended overnight at 4 °C in 0.9% NaCl at 4% of the initial culture volume. For acid preparations the pellets were suspended in PBS at 2% of the initial culture volume.

**Table 1:** **Bacterial strains used in the study**

**Strain**	**Source**
TUV93-0 Δ*fliC*	Lab stock
TUV93-0 Δ*fliC* transformed with pEW7 (*fliC*_*H7*_)	Lab stock
TUV93-0 Δ*fliC fliC* transformed with pAT12	This study
TUV93-0 Δ*fliC fliC* transformed with pAT13	This study
TUV93-0 Δ*fliC fliC* transformed with pAT14	This study
TUV93-0 Δ*fliC fliC* transformed with pAT15	This study
TUV93-0 Δ*fliC fliC* transformed with pAT16	This study

For acid preparation 1 M HCl was added on a stirring platform until a pH ~ 2 was reached and stirred for 30 min. The preparations were centrifuged at 5000 × *g* for 30 min. The supernatants were transferred and neutralised with 1 M NaOH. The volume of supernatants was measured and (NH_4_)_2_SO_4_ added to 2.67 M (35.2 g/100 mL) and left overnight at 4 °C. Then the preparations were centrifuged at 15 000 × *g* at 4 °C for 15 min. The supernatants were discarded and pellets suspended in PBS (2 mL for an original litre of culture). Flagella preparations were dialysed overnight three times at 4 °C in at 500-1000 volumes of PBS and stored at −20 °C.

For sheared flagella preparations, the cultures suspended in a 2% volume of PBS bacterial cultures were made up to 20 mL in PBS. The cultures were sheared for 2 min on ice with an IKA T-10 homogeniser (Ultra-Turrax). The cultures were then centrifuged at 4100 × *g* for 15 min. The supernatants were collected and re-centrifuged. This step was repeated 4 to 5 times until the supernatants were clear. The cleared supernatants were centrifuged at 15 000 × *g* for 10 min to remove any remaining bacteria. The supernatant from this step were transferred into fresh tubes and centrifuged at 145 000 × *g* at 4 °C for 90 min to pellet the flagella. Flagella pellets were suspended in 500 μL PBS and stored at −20 °C.

Flagellin concentrations were determined by BCA and purity by coomassie staining following separation on SDS-polyacrylamide gels.

### LPS removal and monomerization of flagella

The first step to remove LPS involved repeated extraction in 1% TritonX114. This was added to flagella (1 in 500) in an Eppendorf and rotated overnight at 4 °C. The mixture was then incubated at 37 °C for 10 min and centrifuged for 15 min at 13 000 rpm in desktop centrifuge at RT. The aqueous phase was collected from clearly visible Tx114 at bottom of tube. This extraction was repeated 4 times and the collected flagellin filter-sterilized and dialyzed to remove any remaining of detergent. The second step was a column clean up using Pierce® High-Capacity Endotoxin binding resin following manufacturer’s instructions (Thermo Scientific). Endotoxin levels were measured using an EndoLISA kit according to manufacturer’s instructions (Hyglos GmbH) and expressed as endotoxin units (EU) per μg protein.

When required, monomerisation of flagellins was carried out by heating flagella at 70 °C for 15 min followed by filtration by centrifugation at 5000 × *g* at 4 °C for 30 min using 100 kDa 4 mL filter units (Millipore) [13].

### Site-directed mutagenesis of H7 flagella

L500A/I505A, Q89A, and R90A mutations were selected based on effects previously demonstrated for *Salmonella* FliC [13,18] and Q89D and R90T mutations were selected based on naturally-acquired alleles demonstrated to reduce activation of TLR5 in *Helicobacter pylori* [18]. Site directed mutants were made following the manufacturer’s protocols (QuikChange, Agilent technologies). The primers used for these reactions are in Table 2. Amplified and treated constructs were transformed into *E. coli* XL10-Gold Ultra competent bacteria and plated on LB–ampicillin plates and incubated overnight on 37 °C. Plasmids were extracted and sequenced (DNA Sequencing and Bioinformatics - GATC Biotech, Germany). Selection of required alleles followed sequence analysis using Lazergene software.

**Table 2:** **Primers used for H7-FliC mutagenesis**

**SDM**	**Orientation**	**Oligonucleotide sequence (5’ – 3’)**
L500A/I504A	F	CCCGCTTGCTGCCGCGGACGACGCAGCCAGCTCCATCGAC
	R	GTCGATGGAGCTGGCTGCGTCGTCCGCGGCAGCAAGCGGG
Q89A	F	CAACAACAACTTAGCGCGTATTCGTGAAC
	R	GTTCACGAATACGCGCTAAGTTGTTGTTG
Q89D	F	CAACAACAACTTAGACCGTATTCGTGAAC
	R	GTTCACGAATACGGTCTAAGTTGTTGTTG
R90A	F	CAACAACTTACAGGCTATTCGTGAACTGAC
	R	GTCAGTTCACGAATAGCCTGTAAGTTGTTG
R90A	F	CAACAACTTACAGACTATTCGTGAACTGAC
	R	GTCAGTTCACGAATAGTCTGTAAGTTGTTG

### Motility assay

TUV93-0 Δ*fliC* transformed with pEW7 (*fliC*_*H7*_) and altered alleles: pAT12 (Q89A), pAT13 (Q89D), pAT14 (R90A), pAT15 (R90T) and pAT16 (L500A/I504A) (Table 1) were stab inoculated into the centre of motility agar plates [18] (0.3% agar, with ampicillin and 1 mM final concentration of IPTG). The plates were incubated at 30 °C overnight and then examined for the extent of spread.

### Cell lines

Embryonic bovine lung (EBL) cells were from laboratory stocks. The cells were grown at 37 °C in a 5% CO_2_ and 80% humidity in Dulbecco’s modified Eagle’s medium (DMEM) supplemented with 10% (v/v) (FBS, Sigma), 1 U of penicillin (Invitrogen), 1 μg/mL of streptomycin (Invitrogen) and 2 mM L-glutamine (Invitrogen). HEK293 and HEK blue (PDRIVE5s-mIFN-ß) cells were grown in the above medium with additions when required zeocin 100 μg/mL (InvivoGen), pUNo1 selection blasticidin 30 μg/mL (InvivoGen) and Normocin (100 μg/mL) (InvivoGen) was used as antifungal and mycoplasma agent.

### TLR5 clones and transfections

The human and bovine TLR5 clones were obtained from InvivoGen. The human clone was supplied in embryonic kidney cells (HEK293) transfected with pUNo1-hTLR05 (InvivoGen) under control of a hEF1-HTLV promoter. These cells were also transfected with PDRIVE5s-mIFN-ß containing SEAP as a reporter for NF-κB transcriptional activation as the mIFNß promoter has four binding sites for NF-κB. Cells containing the two plasmids are referred to as “hHEK blue cells” while those just the SEAP construct are referred to as “Null cells”. Confluent cultured (HEK blue cells) were used to study the capacity of H7 flagellin to activate hTLR5. The bovine clone was supplied by InvivoGen in *E. coli*, and so was purified and transfected into the HEK Null cells. Permanent transfection was selected by addition of zeocin for the SEAP reporter plasmid and Blasticidin for the bTLR5 clone.

For transfection of the bovine EBLs, the bTLR5 clone was sub-cloned into a modified ptGFP1 vector [26]. Briefly, the full-length bTLR5 was amplified using specific primers encoding bovine TLR5 alongside restriction enzymes sites XhoI and SacII (bTLR5xsFW: CTCGAGCAC CATGGGAGACTGCCTTG, bTLR5xsRV: CCGCGGCTAGGAGATGGTGG). Invivogen bTLR5 plasmid was used as a template in a 25 μL reaction containing 1 μL of plasmid, 5 μL of GoTaq colourless reaction buffer, 0.5 μL of each of the primers at 10 mM, 0.5 μL dNTP (10 mM each), 0.25 μL of a mix of 10:1 GoTaq DNA polymerase (5 U/μL) and Pfu DNA polymerase (5 U/mL) (both Promega, Madison, USA). The PCR product was then gel extracted using QIAquick Gel Extraction Kit (Qiagen Inc., Netherlands) and ligated into pGEM®-T Easy Cloning Vector (Promega, Madison, USA).

Following transformation into competent XL1-Blue Competent Cells (Stratagene, Agilent Technologies Division, USA), cells were grown on LB agar (Sigma Aldrich, USA) supplemented with X-Gal and 10 mM IPTG. White colonies were grown overnight in 5 mL of LB medium with ampicillin (100 μg/mL), in a shaking incubator at 37 °C. Plasmid DNA from 4 independent colonies was then purified using a QIAprep Plasmid DNA Miniprep kit (Qiagen Inc., Netherlands) following the manufacturer’s instructions and sent for sequencing to confirm correct sequence. The confirmed bTLR5 plasmid and modified vector ptGFP1 were then digested with XhoI and SacII at 37 °C for 3 h in a 50 μL reaction. Digestion was then purified with a QIAquick PCR Purification kit (Qiagen Inc., Netherlands) and ligated at 4 °C overnight using T4 DNA ligase (Promega, Madison, USA). Ligated products were then transformed into competent JM109 cells (Promega, Madison, USA), and seeded into LB agar + kanamycin (50 μg/mL). Positive colonies were grown overnight in 5 mL of LB medium with kanamycin in a shaking incubator at 37 °C. Plasmid DNA was purified as above and concentrations determined by spectrophotometry (Nanodrop, Labtech International, UK).

EBL cells were incubated in DMEM media supplemented with 10% foetal calf serum (FCS, Labtech International, UK), 100 U/mL penicillin and 100 mg/mL streptomycin (P/S, Invitrogen Life Technologies, BV, Netherlands) and 1% L-Glutamine. Cells were passaged 48 h prior to transfection by electroporation in a Nucleofector™ 2d Device (Lonza Group Ltd., USA) as described elsewhere [27]. Briefly approximately 2 μg of bTLR5-ptGFP1 construct or empty ptGFP1 vector (control) were used to transfect 10^6^ cells alongside 100 μL Amaxa Nucleofactor solution V, by running program T-030. 500 μl of DMEM, 10% FCS, 1% L-Glutamine and P/S was added to the electrophoresis cuvette and then transferred into tissue culture flasks or plates. After 24 h, all media was replaced with fresh media supplemented with 500 μg/mL of neomycin (G418, Sigma Aldrich, UK). This was replenished every day to remove dead cells, and the concentration of neomycin reduced to 250 μg/mL a week after transfection. Once around 25-50% of the cells were GFP positive, they were cell sorted using a FACSAria™ III which generated a culture with >95% GFP+ cells.

In order to further confirm the presence of bTLR5 in the transfected cells, a PCR was carried out using cDNA obtained from EBL cells transfected with bTLR5-ptGFP1 and empty ptGFP1 vector as template. Forward primer was specific for bTLR5 (bTLR5FW: GTGCCTCGAAGC CTTCAGTTAT) and reverse primer (bTLR5-ptGFP1-RV: CTAGCCGCGGCTCTAGATCAT AATCAG) was designed spanning both bTLR5 and ptGFP1 to ascertain that the amplified fragment was the transfected bTLR5 and not any endogenous TLR5 which could be naturally produced by EBL cells. The reactions for the amplification of the vector/bTLR5 and the endogenous control GAPDH (GAPDH-FW: GATGCTGGTGCTGAGTATGTAGTG and GAPDH-RV: ATCCACAACAGACACGTTGGGAG) was subjected to an initial denaturation of 2 min at 95 °C, followed by 35 cycles of 95 °C for 30 s, 60 °C for 30 s and 72 °C for 45 s using a 25 μL reaction as detailed previously. All PCR products were visualised on a 1% agarose gel containing SYBR® Safe DNA Gel Stain (Life Technologies, BV, Netherlands).

### Bovine monocyte-derived macrophages

Bovine monocyte-derived macrophages (MDM) were generated as described previously [28,29], except that the MDM were differentiated for 14 days. In brief, PBMC were initially cultured for 2 h in RPMI-1640 medium without serum at 5 × 10^6^ cells/mL, before the medium was replaced with MDM medium (RPMI-1640 supplemented with 20% FBS, 4 mM L-glutamine and 50 μM β-mercaptoethanol) with 100 U/mL Penicillin-Streptomycin. The MDM medium was replaced on days 4 and 11. On day 14 the adherent cells were rigorously washed with phosphate buffered saline (PBS) and detached with TrypLE Express (Invitrogen). Flow cytometry, using a mouse anti-bovine SIRPα antibody directly conjugated with RPE-Cy5 (AbD Serotec: Cat. No. MCA2041C), confirmed that the MDM purity exceeded 90% (data not shown). Purified MDM were resuspended at 3 × 10^5^ cells/mL in MDM medium without Penicillin-Streptomycin, dispensed into 12 well plates and cultured for 24 h before transfection of siRNA.

### Analysis of NfκB activation using a secreted alkaline phosphatase reporter

Cells were treated with trypsin and diluted to be 5 × 10^5^ cell per mL. 180 μL/well of the cells were added to 96 well plate and 20 μL of the required flagella concentration added and incubated with the cells overnight at 37 °C in a 5% CO_2_ and 80% humidity. Next day 20 μL of the culture media was added to 200 μL of a ready-to-use substrate (Quanti blue) in a 96 well plate format (p7998, Sigma, St. Louis, MI, USA) for 10 min at RT and the readings made at 650 nm using Promega plate reader.

### CXCL8/IL8 determination by ELISA

Cells were treated as above and CXCL8/IL8 levels in the cell culture supernatants was assessed following capture with mouse anti-sheep IL-8 and detection with rabbit anti-sheep IL-8 (AbD Serotec, Raleigh, N.C.) followed by goat anti-rabbit IgG conjugated to HRP (DAKO) according to manufacturer’s instructions. Briefly, ELISA plates (Thermoelectron 3455) were coated with mouse anti-sheep CXCL8/IL8 antibody (MCA1660) diluted in carbonate coating buffer (50 μL per well). The plates were sealed and incubated at 4 °C overnight. The plates were washed 6 times in wash buffer (0.05% Triton in PBS) and 100 μL/well blocking buffer (3% bovine serum albumen in wash buffer) was added and incubated at RT for 1 h. Samples and standards were prepared as appropriate in dilution buffer (wash buffer + 1% BSA). Plates were washed 5 times in wash buffer and 50 μL of sample and standard were added in duplicate to the plates. The plates were incubated at RT for 1 h. The plates were then washed 6 times and 50 μL/well of the rabbit anti-sheep IL-8 antibody (AHP425) diluted in dilution buffer was added. The plates were then incubated at RT for 1 h. The plates washed 6 times and 50 μL/well of goat anti-rabbit-HRP (Dako P0448) diluted 1:1000 in RDB was added and incubated at RT for 1 h. The plates were wash 6 times and 100 μL OPD substrate (Sigmafast OPD P9187 Sigma-Aldrich) was added per well and incubated for 5-10 min depending on reaction time of the controls and this varied depending on the room temperature. The reactions were stopped by addition of 50 μL 2.5 M H_2_SO_4_ and plates were read at 492 nm using a BMG FLUORstar ELISA reader. The concentration of CXCL8 was determined according to manufacturer’s instructions using recombinant bovine CXCL8 (Kingfisher Biotech, Inc*.*) to make the standard curve.

We note that sets of experiments were performed with EBL cells at different densities and transfection efficiencies which, in combination with different flagellin preparations may account for variation in CXCL8 levels measured at specific flagellin concentrations between experiments.

### Real time PCR analysis of TLR5 and CXCL8 transcripts

Total RNA was extracted from all MDM samples using the RNeasy mini kit (Qiagen), including a DNase digestion step, according to the manufacturer’s instructions. The quality and quantity of the resulting RNA was determined by a Nanodrop spectrophotometer. First strand cDNA was reverse transcribed from 0.2 μg total RNA using an oligo(dT) primer and GoScript reverse transcriptase (Promega) according to the manufacturer’s protocols. The resulting cDNA was diluted 1:20 for RT-qPCR analysis.

Oligonucleotides were designed for bovine TLR5, IL1β and CXCL8/IL8 using Primer3 [30] and Netprimer (Biosoft International) software (Table 3). The mRNA levels of each transcript were quantified by qPCR using the Brilliant III ultra-fast SYBR Green Mastermix kit (Agilent) as described previously [28]. The relative quantities of mRNA were calculated using the method described by Pfaffl [31]. The RT-qPCR results for chromosome alignment maintaining phosphoprotein 1 (CHAMP1) were used to calculate differences in the template RNA levels and thereby standardize the results for the genes of interest. CHAMP1 was previously selected from microarray and RT-qPCR analyses as a constitutively and moderately expressed gene in activated bovine monocytes and macrophages [28,29].

**Table 3:** **Additional primers used in the study**

**Gene**	**Accession no.**	**Orientation**	**Oligonucleotide sequence (5’ – 3’)**
Toll-like receptor 5 (TLR5)	NM_001040501	F	CGATGCCTATTTGTGCTTCA
		R	CACCACCCGTCTCTAAGGAA
Interleukin 1B (IL1B)	NM_174093	F	TCCGACGAGTTTCTGTGTGA
		R	TGTGAGAGGAGGTGGAGAGC
Interleukin/CXCL 8 (IL8/CXCL8)	NM_173925	F	CACATTCCACACCTTTCCAC
		R	GGCAGACCTCGTTTCCATT
Chromosome alignment	NM_001205506	F	AGCAGTGACCAAGAGCAGGT
Maintaining phosphoprotein 1		R	TCATAGCACGACAGCAACAA

F and R denote forward and reverse primers respectively.

### siRNA knock-down of TLR5

Prior to the experiments reported here, three siRNA duplexes specific for bovine TLR5 (RefSeq Accession No. NM_001040501) were obtained from Sigma-Aldrich and assessed for their ability to knockdown TLR5 mRNA levels. The siRNAs TLR5#2 (target sequence CAATTTCATCCAATTATCA) and TLR5#3(target sequence CGTACAAATACGATGCCUA) were found to consistently knock-down TLR5 mRNA levels between 24 and 96 h. The third siRNA TLR5#1 (target sequence GTCTGAACCCATTCAGAAA) failed to modulate TLR5 mRNA levels. In addition, the AllStars negative control siRNA, which does not share homology with any known mammalian gene (Qiagen), was used as a non-target siRNA control.

The transfection reagent Lipofectamine RNAiMAX (Invitrogen) has previously been shown to be suitable for transfecting siRNA into bovine MDM [28]. The MDM were transfected with siRNA following the manufacturer’s protocol, initially generating a mix of 3 μL Lipofectamine RNAiMAX and 3 μL 20μM siRNA in 200 μL Opti-MEM I reduced serum medium (Invitrogen). After 20 min incubation at RT the siRNA/Lipofectamine RNAiMAX mix was added to MDM in 1 mL MDM medium, giving a final concentration of 50 μM siRNA. Additional controls included in each experiment were MDM treated with Lipofectamine RNAiMAX only (transfection control) and untreated MDM (negative control). After 24 h the medium was replaced with fresh MDM medium to remove the residual siRNA/Lipofectamine RNAiMAX mix.

### Immunization of cattle

Immunizations were performed at Moredun Research Institute (MRI) under Home Office licence 60/3179. Ethical approval was obtained from the MRI Animal Experiments Committee. Two groups of conventionally reared male Holstein–Friesian calves (*n* = 6) were immunized on two separate occasions two weeks apart with either 60 μg of WT-H7 + 5mg Quil A adjuvant (Brenntag Biosector, Frederikssund, Denmark) or 60 μg R90T-H7 + 5 mg Quil A adjuvant via the intra-muscular route. A control group (*n* = 3) was immunized in an identical manner with adjuvant only. Serum samples were collected at days -1, 7, 21 and 28 relative to the first immunization. The average age of calves at the time of the first immunization was 14 ± 3 weeks. Faecal samples obtained from each calf prior to immunization were confirmed to be negative for *E. coli* O157:H7 by immunomagnetic separation, performed according to the manufacturer’s instructions (Dynabeads® anti-*E. coli* O157, Invitrogen).

### Measurement of serum immunoglobulin levels

WT-H7 and R90T-H7 specific IgA antibodies were quantified by indirect ELISA as previously described [8] using LPS-free antigen preparations. Antibody titres were calculated as follows: the log_10_ optical density (OD) was plotted against the log_10_ sample dilution and regression analysis of the linear part of the curve allowed the calculation of the endpoint titre with an OD of 0.1 above the average negative control value. Inter-plate variation was normalised to a known positive control sample.

### Statistical analyses

Mixed models were used to analyse each experiment. These models are an extension to ANOVA and take account of variation occurring both between- and within- experiments, and of correlations between measurements repeated over time-points or concentrations. Treatment, concentration (or time) and the treatment by concentration (or time) interaction were fitted as fixed effects, and experiment as a random effect, in each model. Pairwise comparisons between treatment effects at individual concentration (or time) levels were made within each model using t-tests. The variance for these comparisons was determined by the mixed model, taking into account the between- and within- experiment variation. Adjustments were made for multiple testing in the statistical tests. When measurements were not repeated over concentration or time, only treatment was fitted as a fixed effect in the mixed model. Log transformations of the data were used when necessary to ensure that normality requirements for the model were satisfied. The analyses were carried out using the MIXED procedure in the SAS/STAT(r) software. Further information on mixed models may be found in Brown and Prescott [32].

## Results

### Signalling from HEK293 cells transfected with human and bovine TLR5 clones

**Figure 1:**
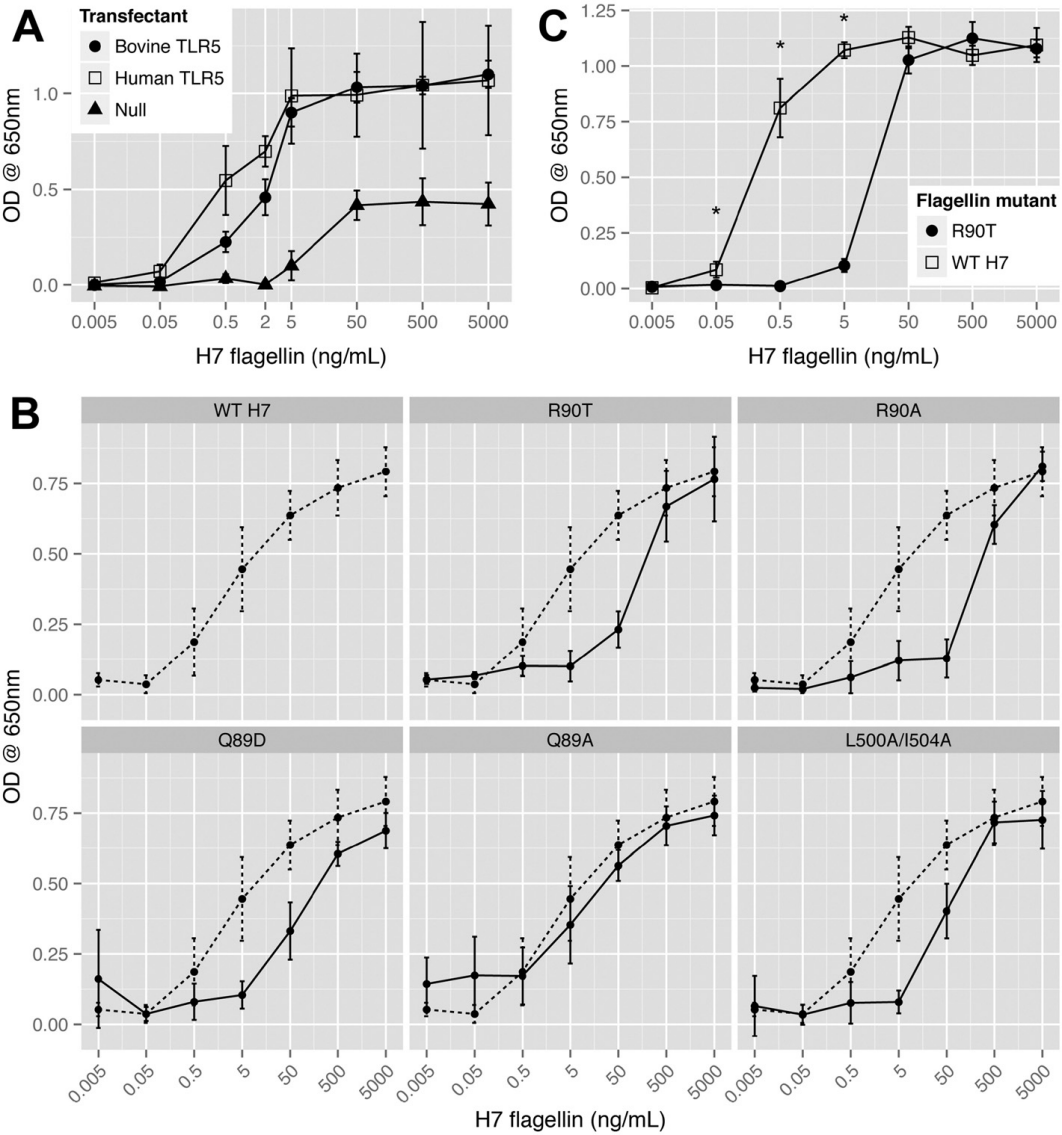
**NF-kB dependent signalling from human and bovine TLR5 clones in HEK293 cells.** HEK293 cells were transfected with an NF-κB-dependent secreted alkaline phosphatase (SEAP) reporter plasmid alone (Null cells) or with the reporter in combination with either a human TLR5 (hTLR5) or bovine (bTLR5) expression plasmid. SEAP levels were determined following addition of different concentrations of WT-H7 flagellin or site-directed flagellin mutants. **(A)** NF-kB dependent SEAP production from bTLR5 and hTLR5 following overnight stimulation with a range of H7 flagellin concentrations. SEAP activity from cells transfected with either TLR5 construct was significantly higher than the non-transfected control at levels of 0.5 ng/mL flagellin or greater (*p* < 0.005). hTLR5 produced higher SEAP activity compared to bTLR5 at two concentrations of flagellin, 0.05 and 0.5 ng/mL (*p* = 0.027 and *p* < 0.001 respectively). **(B)** NF-kB dependent SEAP production from bTLR5 in HEK293 cells stimulated overnight with different concentrations of WT-H7 and the indicated flagellin mutants. The dashed line indicates the response to WT-H7. **(C)** NF-kB dependent SEAP production from hTLR5 in HEK293 cells following addition of WT H7 and an R90T variant. Differences of *p* < 0.001 are indicated by an asterisk. All experiments in this figure were performed a minimum of three times with at least three technical repeats. The data shown are the means and 95% confidence intervals.

The bovine TLR5 (bTLR5) and human TLR5 (hTLR5) clones were obtained from InvivoGen. As a read-out for activity of HEK293 cells transfected with these constructs, the cells also contained a plasmid encoding an NF-κB/AP-1-inducible secreted alkaline phosphatase (SEAP) reporter (Materials and methods, InvivoGen). HEK293 cells stably transfected with just the reporter construct but no TLR5 clones were used as a control (Null cells). Both the bTLR5 and hTLR5 clones activated the NF-kB reporter at levels significantly higher (*p* < 0.005) than the Null cells over a range of H7 flagellin concentrations (0.5-5000 ng/mL, Figure 1A). There was evidence that hTLR5 was more sensitive than bTLR5 to activation by flagellin at lower concentrations (0.05 and 0.5 ng/mL, *p* = 0.027 and *p* < 0.001 respectively), but differences in expression levels between the two constructs cannot be ruled out. There was no significant difference in their maximum levels of induction above a concentration of 5 ng/mL flagellin. This indicated that the bTLR5 was capable of signalling through NF-kB in this human-derived cell background.

### Mutational analysis of H7 flagellin

**Figure 2:**
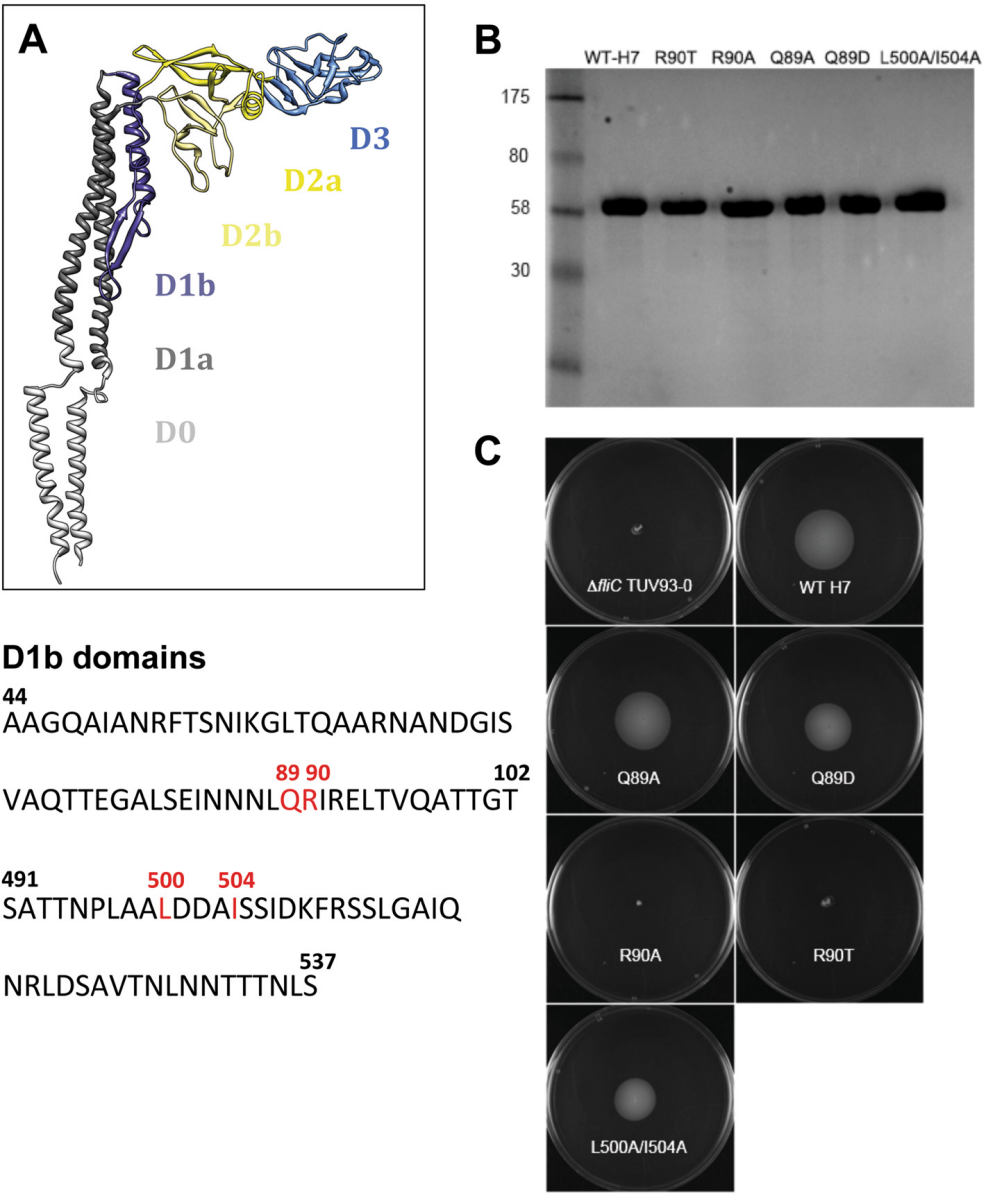
**Site-directed mutagenesis of H7 flagellin to reduce TLR5 signalling. (A)** Predicted TLR5-binding regions of H7 flagellin (FliC). Top panel shows the structure of FliC (*Salmonella* Typhimurium) from PDB entry 1UCU (R-type straight flagellar filament) and is coloured in UCSF Chimera according to structural domains as indicated. TLR5 binding residues have been mapped within the D1b regions (dark grey). The bottom panel shows the FliC-H7 D1b regions which are homologous to those from *S*. Typhimurium and the 4 residues that have been mutated in this study are shown in red. Q89 and R90 in the D1b amino terminal regions have the same position in *S*. Typhimurium, whereas the L500 and I504 in FliC-H7 are equivalent to I411 and L415 (leucine and isoleucine are interchanged) respectively in *S*. Typhimurium phase 1 FliC (13). **(B)** Coomassie-stained SDS-PAGE of WT-H7 and variants. Flagella were purified from *E. coli* O157 TUV93-0 Δ*fliC* and total protein concentration adjusted following a BCA assay to 2.5 μg per lane. The left margin shows the approximate molecular size (kDa). **(C)** Motility of *E. coli* O157 expressing altered flagellins. Motility was assessed following inoculation of *E. coli* O157 (TUV93-0) Δ*fliC* containing WT *fliC*_*H7*_ clone (pEW7) and site-directed mutants as indicated. Motility was assessed after overnight incubation.

Previous mutational analyses of *Salmonella enterica* serovar Typhimurium Phase 1 FliC flagellin has helped define key amino acids required for TLR5 recognition and signalling within the TLR5 binding domains in the D1 regions of the flagellin monomer (Figure 2A) [13]. We mutated amino acid residues based on this previous work as well as analysis of *Helicobacter* FlaA and FlaB which have been shown to have a reduced capacity to stimulate TLR5 [18] as part of a proposed immune evasion strategy. Five mutations (Figure 2A) were introduced successfully into a clone of *fliC*_*H7*_ on plasmid pEW7 (Materials and methods). Flagella expression was analysed in *E. coli* O157 (TUV93-0) Δ*fliC* transformed with the WT *fliC*_*H7*_ or the site-directed mutants. Flagella were purified from all the mutants by shearing and concentrations were adjusted based on bicinchoninic acid assays and then analysed by gel electrophoresis for purity and relative concentration (Figure 2B). The Q89A, Q89D, L500A/I504A demonstrated motility on 0.3% agar following overnight culture, whereas R90A-H7 and R90T-H7 were non motile (Figure 2C). The different flagellins were tested over a range of concentrations for their capacity to stimulate NF-kB activity in HEK293 cells containing the bTLR5 clone (Figure 1B). All variants with the exception of Q89A showed reduced SEAP levels at flagellin concentrations of 5 ng/mL and 50 ng/mL (*p* < 0.001). R90T was selected for further work as this could easily be purified at high levels and showed a markedly reduced signalling capacity compared to WT-H7. R90T was then compared against the WT-H7 using the hTLR5 clone in HEK293 cells and analysis demonstrated significantly reduced NF-kB dependent SEAP production over a range of flagellin concentrations (0.005-5 ng/mL, *p* < 0.001), with approximately 100-fold more mutated flagellin (R90T) required to induce equivalent levels of response to the WT monomer at these points on the response curves (Figure 1C).

### Analysis of bovine TLR5 activity in bovine epithelial cells

There are possible complications with using the HEK293 cell background to read out for functionality of bTLR5. These include that the adaptor signalling pathways are human not bovine and the possibility of some complementing activity from residual TLR5 activity in the HEK293 cells [17,33], as our non-transfected cells did show a level of activation, especially at high flagellin concentrations (Figure 1A). It was important therefore to test the activity of the bTLR5 clone in a bovine cell context. To our knowledge there are two main immortalized bovine epithelial cell lines that are currently used by research community: Mac-T, a bovine mammary epithelial cell line [34] and EBLs, an embryonic bovine epithelial cell line (supplied by the Leibniz Institute DSMZ-German Collection of Microorganisms and Cell Cultures). We use EBLs routinely for bacterial infections studies so we investigated whether these cells were able to produce CXCL8 following addition of H7 flagellin. There was no detectable endogenous TLR5 activity in these cells as determined by CXCL8 assays 16 h after addition of up to 10 μg of flagellin (data not shown) and so they were considered an appropriate background in which to study the bTLR5 clone. In order to aid detection and selection of transfected EBLs, the bTLR5 region was sub-cloned into ptGFP1 (Material and Methods) allowing detection of transfected cells through expression of GFP.

**Figure 3:**
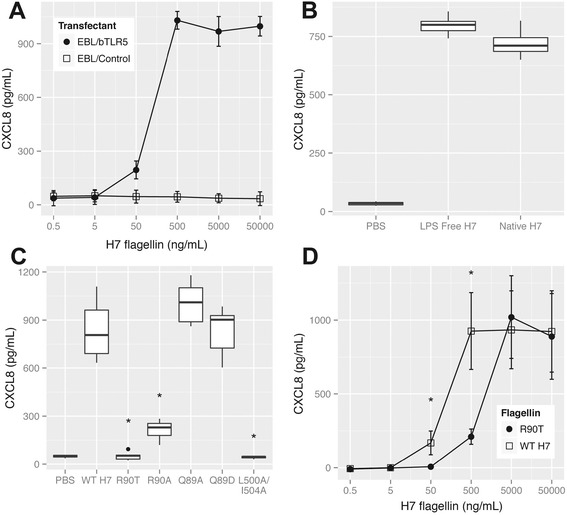
**Analysis of bovine TLR5 activity in bovine epithelial cells.** The bovine EBL cell line was stably transfected with a bTLR5 clone and CXCL8 secretion assayed following challenge with flagellin. **(A)** CXCL8 levels from EBLs with and without transfection of bTLR5. H7 flagellin at a range of concentrations was added to the cells and CXCL8 levels were measured by ELISA following overnight incubation. Transfection with bTLR5 resulted in significantly higher levels of CXCL8 being produced by the bTLR5+ cells with addition of 50-50 000 ng/mL of H7 flagellin (*p* < 0.001). The data shown are the means and 95% confidence intervals. **(B)** Analysis of CXCL8 production from EBL-TLR5 in response to addition of native H7 or an H7 flagellin preparation from which the majority of LPS has been removed. Medians and interquartile ranges are shown. **(C)** Secreted CXCL8 following addition of WT and mutated H7 flagellins to EBLs with transfected bTLR5. 50 ng/mL of WT-H7 and mutated flagellins were added to the EBLs transfected with bTLR5 and incubated overnight. CXCL8 was measured by ELISA. Addition of the R90T, R90A and L500A/I504A variants led to significantly lower levels of cytokine release (*p* < 0.001) relative to WT-H7 stimulation (asterisks). R90T showed a significantly lower induction than R90A (*p* < 0.001). Medians and interquartile ranges are shown. **(D)** Secreted CXCL8 following addition of WT H7 and the R90T flagellin mutant to EBL cells. A range of flagellin concentrations was incubated overnight with the cells and CXCL8 measured by ELISA. R90T flagellin demonstrated significantly reduced levels of CXCL8 activation at 50 ng/mL and 500 ng/mL (*p* < 0.001), marked by asterisks. The data shown are the means and 95% confidence intervals. All CXCL8 data shown is from a minimum of three biological replicates.

EBL cells with and without sub-cloned bTLR5 were then tested for CXCL8 secretion following addition of flagellin (0.5 ng/mL - 50 μg/mL). The results indicated that transfecting these cells with the bTLR5 clone enabled them to respond to the addition of flagellin by releasing CXCL8 over a range of flagellin concentrations (Figure 3A, *p* < 0.001 compared to control cells). Induction of CXCL8 by LPS was ruled out in two ways; (1) the majority of LPS was removed from an H7 flagellin preparation (Materials and methods) leaving less that 0.05 endotoxin units/μg protein (EU) and this still was able to promote CXCL8 production to a level equivalent to the original (LPS high) preparation (Figure 3B). Secondly, addition of purified LPS (1 EU) alone failed to induce any CXCL8 production from EBLs transfected with bTLR5 (data not shown).

The site-directed mutations in the TLR5 binding domain of FliC_H7_ were tested at 50 ng/mL on EBL cells transfected with bTLR5 (Figure 3C). The R90T, R90A and L500A/I504A constructs all showed significantly reduced levels of CXCL8 secretion compared to stimulation with WT-H7 (*p* < 0.001). At this flagellin concentration R90T produced lower levels of CXCL8 than the R90A construct (Figure 3C, *p* < 0.001). Of note was the finding that neither Q89A nor Q89D had any impact on levels of CXCL8 produced compared to WT-H7 at this flagellin concentration while this was not the case when these were tested on the hTLR5 in the HEK293 cells, indicative of different recognition or signalling between the clones and cell types. The R90T flagellin was compared with WT-H7 over a range of concentrations on the bTLR5-EBL cells and shown to induce significantly lower levels of CXCL8 when 50 ng/mL and 500 ng/mL flagellin was added (Figure 3D, *p* < 0.001). At these concentrations approximately 10-fold more R90T than WT was required to induce an equivalent level of CXCL8 release (Figure 3D).

### Analysis of TLR5 responses in bovine primary macrophages derived from PBMCs

**Figure 4:**
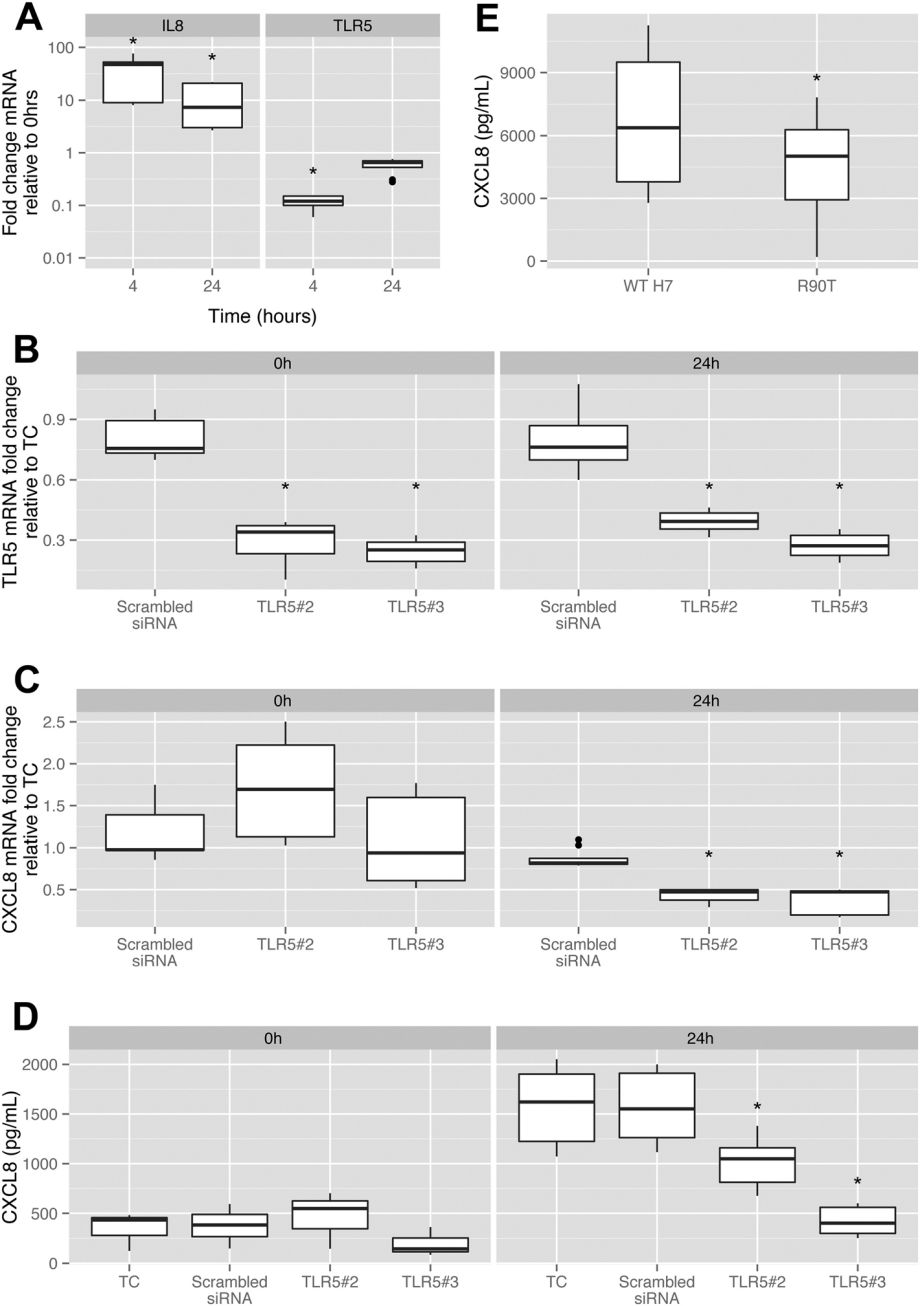
**Responses of bovine monocyte-derived macrophages (bMDMs) to flagellin. (A)** mRNA levels of CXCL8 and TLR5 following addition of flagellin to bMDMs. Transcript levels are plotted relative to time 0 and increase significantly at 4 h and 24 h (*p* < 0.001), while TLR5 transcript levels are lower (*p* < 0.001) 4 h after addition of flagellin but not significantly different (*p* = 0.073) at 24 h, significance marked by asterisks. **(B)** Assessment of TLR5 transcript levels in bMDMs. Transfected cells were pre-incubated with siRNAs for 48 h then WT H7 flagellin (26.5 ng/mL) added (time 0). TLR5 transcripts were reduced by treatment with both TLR5#2 and TLR5#3 relative to controls at both time 0 and 24 h (*p* < 0.001), marked by asterisks. **(C)** Assessment of CXCL8 transcript levels in bMDMs. CXCL8 mRNA levels were measured in cells challenged with WT H7 flagellin as above. CXCL8 transcript levels were significantly reduced in the cells treated with the TLR5#2 and TLR5#3 siRNAs relative to the controls at 24 h (*p* < 0.001), indicated by asterisks. Data is from three biological repeats with a minimum of three technical replicates. **(D)** Determination of released CXCL8 from bMDMs with reduced TLR5 expression. bMDMs were treated as above and supernatant CXCL8 determined by ELISA. TLR5 #3 siRNA significantly reduced secreted levels of CXCL8 relative to un-transfected controls at 24 h (*p* < 0.001), while a significant reduction was evident for TLR5 #2 at 24 h (*p* = 0.012), marked with asterisks. **(E)** CXCL8 release from bMDMs challenged with WT and altered H7 flagellin. CXCL8 was measured in the supernatants of the bMDMs 24 h after stimulation with WT or R90T H7 flagellin. The CXCL8 levels released were significantly lower for R90T compared with WT flagellin (*p* = 0.022, asterisk). All plots show medians with upper and lower quartiles; outliers as dark circles.

We wanted to determine if CXCL8 responses from bovine peripheral blood monocyte-derived macrophages (MDMs) exposed to flagellin were TLR5-dependent. MDMs were derived as described previously ([28,29] and Materials and methods). To determine the initial response of the bMDMs to flagellin, TLR5 and CXCL8 transcript levels were determined at 4 and 24 h by RT-PCR following addition of 26.5 ng/mL H7 flagellin (Figure 4A). CXCL8 mRNA levels increased at both time points relative to the T0 value (*p* < 0.001) while TLR5 levels were significantly reduced at 4 h (*p* < 0.001) while there was no significant difference at 24 h (*p* = 0.073, Figure 4A).

In order to assess the requirement of TLR5 for CXCL8 production following flagellin stimulation, three different siRNAs were tested to try and reduce TLR5 transcript levels in the bovine MDMs using established methods [28]. The bovine MDMs were cultured for 48 h following siRNA transfection. TLR5 and CXCL8 mRNA levels are shown at 0 and 24 h following addition of 26.5 ng/mL flagellin (Figures 4B and 4C). It was evident that two of the TLR5 siRNAs, #2 & #3, had significantly reduced levels of TLR5 mRNA at 24 h post flagellin stimulation relative to both non-transfected and scrambled siRNA controls (*p* < 0.001, Figure 4B). The third siRNA had no effect and is not shown. CXCL8 mRNA levels (Figure 4C) were reduced at 24 h post flagellin addition for the cells transfected with siRNAs #2 & #3 relative to the controls (*p* < 0.05) and this correlated with a significant reduction in secreted CXCL8 for siRNA #2 at 24 h (*p* = 0.012) and for siRNA #3 at 24 h (*p* < 0.001) relative to controls (Figure 4D). Bovine MDMs treated with siRNA #3 produced less CXCL8 in response to crude LPS activation than measured in siRNA #2-treated and control MDMs (data not shown), suggesting another effect of this siRNA on bovine MDM function or viability. This may explain the enhanced effect of siRNA #3 compared to siRNA #2 with respect to CXCL8 mRNA expression and protein secretion. Taken together, these data indicate a positive association of TLR5 expression in bovine MDMs with both CXCL8 mRNA expression and CXCL8 secreted cytokine production in response to addition of H7 flagellin.

To determine if a variant flagellin with reduced TLR5 activation (relative to WT-H7) also had a reduced capacity to stimulate CXCL8 when added to the bovine MDMs, the R90T flagellin was tested. While addition of 50 ng/mL R90T produced significantly lower levels of secreted CXCL8 compared to addition of WT H7 (*p* = 0.02, Figure 4E), this reduction in secreted CXCL8 levels was only 50% compared to the >10 fold difference demonstrated for the same variant on EBLs expressing bTLR5 (Figure 3C).

### Analysis of bovine humoral responses to systemic immunization with WT-H7 flagella and R90T derivative

**Figure 5:**
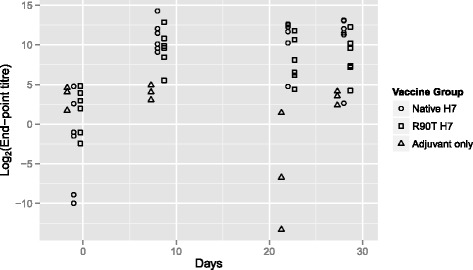
**IgA responses of cattle immunized with WT and R90T H7 flagellin.** Three groups of calves were immunized twice via the intramuscular route 2 weeks apart with either 60 μg of WT H7 flagellin + 5mg Quil A (*n* = 6), 60 μg of R90T H7 flagellin + 5mg Quil A (*n* = 6) or 5mg Quil A alone (*n* = 3) as described in the Materials and methods. Levels of anti WT-H7 specific IgA were determined by end-point ELISA from serum sampled at the times indicated. Animals were immunized at day 0 and day 14. Plots show the log_2_ transformed endpoint titre values for each animal sample for each of the three groups. The sampling times were a day before immunization, and days, 8, 22 and 28 post-immunization; the groups have been separated to ease visualization.

Two groups of male Holstein-Friesian calves (*n* = 6) were immunized twice with an interval of two weeks (day 0 and day 14) with either WT-H7 plus Quil A adjuvant or R90T-H7 plus Quil A adjuvant. A further group of three calves were immunized with adjuvant only. The LPS levels in the two flagellin preparations were equivalent (Materials and Methods). Serum IgA titres against both LPS-free (<0.05 EU per μg protein) WT-H7 and R90T-H7 were determined by end-point ELISA until two weeks after the second immunization (Figure 5). Immunization with both WT-H7 and R90T-H7 resulted in significant increases in serum IgA antibodies against WT-H7 from 7 days after the first immunization and these remained elevated until two weeks after the second immunization by comparison to serum from animals immunized with adjuvant only (Figure 5, *p* < 0.01). The same pattern was observed when IgA responses were analysed with plates coated with R90T-H7 indicating that the amino acid change was unlikely to have altered overall presentation of the flagellin and epitopes (data not shown). There was no significant difference in IgA levels produced between the groups immunized with WT-H7 and R90T-H7, although median levels of IgA from the animals immunized with WT-H7 were consistently higher than those receiving R90T-H7 (Figure 5A). The variation between samples and low animal numbers means that the study was underpowered to detect relatively subtle differences in IgA titres. Combining the T2-T4 IgA responses for the two groups approaches significance (*p* = 0.07) in terms of a reduced IgA response for the animals immunized with R90T.

## Discussion

There is considerable interest in using factors that stimulate pattern recognition receptors (PRRs) as adjuvants. MAMPs are a logical inclusion in vaccine formulations as they stimulate the natural recognition systems allied to host innate and adaptive responses [35,36]. Flagellin monomers are powerful agonists through TLR5 activation as demonstrated in mice and humans [20,37] but their potency to activate TLR5 in cattle is unclear. Our work is focused on responses stimulated by H7 flagellin in cattle for two reasons: (1) generically, as an adjuvant that can stimulate mucosal responses in ruminants; (2) as an antigen to limit EHEC O157:H7 colonisation following vaccination in the reservoir host. Our previous research has demonstrated that systemic immunization with H7 flagella can limit EHEC O157:H7 colonisation possibly by eliciting specific IgA response and high IgG titres [3,8]. The nature of this response was in line with recent findings in mice that have shown the importance of TLR5 in mediating an IgA response to flagellins following systemic injection [20]. However, the responses in cattle were counter to a recent report that indicated minimal activity of cells containing a bovine TLR5 clone [24]. The purpose of the current study was therefore to investigate the functionality of bovine TLR5 using a combination of in vitro and in vivo approaches.

The research presented provides evidence that bovine TLR5 (EMBL ABC68311.1) is functional in terms of flagellin recognition and signalling. The TLR5 clone signalled through an NFkB reporter when transfected into HEK293 cells; the signalling was reduced when specific mutations were introduced into the H7 flagellin TLR5-binding domain. It was apparent that the human clone used in our study did activate the NK-kB reporter to higher levels than the bovine clone in the HEK293 background, at least at when the cells were challenged with lower levels of H7 flagellin. We do not know whether this is due to differences in expression levels of the different TLR5s, and/or signalling activity. Recently reported research on bovine TLR5 by Metcalfe et al. [24] provided no evidence of bTLR signalling in HEK293T cells and we reasoned that this may be due to: (1) either very low levels of transfection and a different reporter system in their study; (2) differences in signalling in the HEK293 and HEK293T cells based on residual human TLR activity. We have found positively selected codons in the bovine TIR domain which may alter its interactions with MyD88 and other signalling molecules and impact on signalling in cells of different animal origin [22]. To ensure compatibility, we tested the bovine TLR5 clone in a bovine cell line. Prior to transfection, the bovine epithelial cell line, EBL, showed no production of IL8/CXCL8 when flagellin was added. This cell line was then transfected with a bTLR5 sub-clone generated in this study and this resulted in cells that secreted CXCL8 in response to addition of flagella in a dose-dependent manner. This secretion was reduced when the mutated H7 flagellin R90T was added. While this data indicated that the bTLR5 clone is active in response to flagellin in a bovine cell background, further work is required to assess which cells and tissues express TLR5 in cattle. It was interesting that un-transfected EBL cells did not express a TLR5 transcript (data not shown) and were not activated by addition of H7 flagellin; this is in line with reports that udder tissue does not express TLR5 [38] and both bovine primary colonocyte cultures and bovine neutrophils were negative for TLR5 expression [39,40]. A key restriction for future work on cell- and tissue-specific expression is the development of reagents that recognise ruminant TLR5, particularly in its native conformation.

Based on current research, the key cells involved in stimulating host responses to flagellins are most likely specific subsets of dendritic cells and/or macrophages [20,21]. We therefore wanted to analyse TLR5-dependent responses to H7 flagellin by bovine macrophages. Macrophages were derived from blood monocytes [29] for which RNA silencing methods have been developed [28]. These cells showed detectable expression of TLR5 mRNA which could be knocked down by various TLR5 specific siRNAs. These cells produced CXCL8 mRNA and cytokine in response to flagella and this response was restricted by the anti-TLR5 siRNAs. It was interesting that the level of TLR5 mRNA reduced significantly 4 h following addition of flagellin, indicating that recognition of the agonist leads to a regulatory shift in the sensing cells. However, this immediate repression appeared to be temporary as the TLR5 transcript levels were similar to the initial levels at 24 h, perhaps indicating some type of homeostatic response to continuous flagellin activation. While this study did demonstrate functional TLR5-dependent signalling by bovine macrophages it was evident from the TLR5 knockdowns and studies with the mutated flagellins that other TLR5-independent responses to the flagellins were occurring which did not appear to be the case for the transfected EBL cells. It has been shown that flagellin recognition can also be mediated through cytosolic NAIP and NLRC4-dependent inflammasomes [41,42]. Thus bovine macrophages, unlike bovine EBLs, presumably internalise flagellin into the cytosol, leading to activation of inflammasomes, but further research is required to confirm if this is the case and the consequences of this activation.

To test whether the reduced capacity to stimulate bTLR5 in vitro might alter the humoral IgA response to flagellin, cattle were immunized with either WT-H7 or R90T-H7 flagellin preparations. IgA responses in cattle serum to both flagellins were determined by end-point titration ELISAs. Immunization with the R90T led to levels of serum IgA that were consistently lower than those detected for the WT-H7 irrespective of the coated flagellin antigen used in the ELISA (WT or R90T). However, these differences were not significant (*P* > 0.05) as a consequence of high titre variation between animals and the relatively small numbers of calves in each group (*n* = 6). The finding that the variant R90T-H7 was also able to stimulate IgA responses was perhaps not surprising given that this variant was still able to activate bTLR5 signalling, although at a reduced level compared to WT-H7. Differences in vitro were dependent on the amount of flagellin used and how these levels translate to the sensitivity in vivo is unknown. Furthermore the difference in terms of CXCL8 secretion from bovine MDMs stimulated with WT-H7 vs R90T-H7, while significantly reduced for the variant, was less than two-fold at the concentration examined (Figure 4E). As discussed above, it is possible that other TLR5-independent signalling pathways may contribute to the IgA response in cattle following systemic immunization with flagellin. Another consideration for the cattle immunizations experiments is that QuilA was used as an adjuvant and immune stimulating complexes (ISCOMS) containing Quil A can help induce IgA responses when delivered mucosally. However, we are aware from our previous work examining systemic delivery of antigens other than flagellin that Quil A does not usually induce IgA responses to these [8,9]. So while we appreciate there may well be synergy between the adjuvants, QuilA and flagellin, flagellin is required for overt IgA responses.

Proof of TLR5-dependent IgA production in cattle would ideally require cattle that do not express functional TLR5. Recent work has shown that TLR5 alleles are present in cattle with stop codons in the ectodomain which are likely to impair function [22]. However their frequencies would indicate that where present, they would probably be heterozygous. Nonetheless a different stop codon mutation in human TLR5 is a dominant negative mutation and its presence as a heterozygote in humans leads to significantly lower natural levels of flagellin-specific IgG and IgA [43]. Thus it may be possible to investigate whether cattle carrying the STOP codon mutants respond less well to systemic immunisation with H7 flagellin. It may also be possible to identify bovine sires carrying these STOP codons and select homozygotes which would be TLR5 negative, allowing more definitive experiments on the role of bovine TLR5 in vivo.

The TIR domain is important for signal transduction for TLR5, although less is known about the TLR5 signaling motifs and pathways for other TLRs. It is intriguing that the bTLR5 is functional given that it possesses a Y798P change compared to other mammalian TLR5 sequences and that Y798L has been shown to negate TLR5 signalling activity in mice [23]. However, the serine at position 805 may also be critical for signalling [44], and this is present in the WT bovine TLR5. Evidence suggests that ruminant TLR5 has undergone adaptive evolution which is still ongoing as SNPs were co-localised with predicted adaptive codons [22]. This would suggest that the interactions between flagellins and bovine TLR5 are drivers of evolutionary changes which may have functional consequences in terms of both infectious disease susceptibility and/or inflammatory disorders as described for humans [43,45]. On-going work is investigating the critical residues required for bTLR5 responses and the pathways induced by flagellin recognition. Potentially polymorphisms might account for variation seen in the responses in the flagellin immunisation experiments carried out in the present animal study.

Flores-Langarica et al. [20] have shown in mice that CD103+ TLR5+ dendritic cell numbers drop in the lamina propria following systemic immunization with flagellins, and that this reduction is associated with an increase in these cells within the local mesenteric lymph nodes. They conclude that this subset of cells traffics to the local node following TLR5-dependent flagellin stimulation to present flagellin peptides to T cells and promote class switching of flagellin-specific B cells to IgA. It has been also been shown that humoral immunity in response to flagellin can depend on other TLR5 independent pathways which involve NAIPs and NLRC4 inflammasome activation [27] and/or a novel MyD88 independent pathway [46]. Their relevance for cattle is unknown at present. A logical progression of the work in this area is to obtain similar mucosal responses to systemically delivered heterologous antigens expressed either within or conjugated to flagellins. Future work will test immune responses in cattle induced by heterologous flagellin-antigen constructs, as well as defining the specificity of bovine TLR5 stimulation by different flagellin sequences to determine the most appropriate flagellin constructs for use as an adjuvant in this species.
